# Carboplatin-resistance-related DNA damage repair prognostic gene signature and its association with immune infiltration in breast cancer

**DOI:** 10.3389/fimmu.2025.1522149

**Published:** 2025-01-29

**Authors:** Shuwen Dong, Anqi Li, Ruixin Pan, Jin Hong, Zheng Wang, Kunwei Shen

**Affiliations:** ^1^ Department of General Surgery, Comprehensive Breast Health Center, Ruijin Hospital, Shanghai Jiao Tong University School of Medicine, Shanghai, China; ^2^ Department of Pathology, Ruijin Hospital, Shanghai Jiao Tong University School of Medicine, Shanghai, China

**Keywords:** breast cancer, carboplatin resistance, DNA repair genes, immune infiltration, TONSL

## Abstract

**Introduction:**

Breast cancer is among the most prevalent malignant tumors globally, with carboplatin serving as a standard treatment option. However, resistance often compromises its efficacy. DNA damage repair (DDR) pathways are crucial in determining responses to treatment and are also associated with immune infiltration. This study aimed to identify the DDR genes involved in carboplatin resistance and to elucidate their effects on prognosis, immune infiltration, and drug sensitivity in breast cancer patients.

**Methods:**

A 3D-culture model resistant to carboplatin was constructed and sequenced. Co-expressed DDR genes were analyzed to develop a predictive model. Immune infiltration analysis tools were employed to assess the immune microenvironment of patients with varying expression levels of these risk genes. Additionally, drug sensitivity predictions were made to evaluate the efficacy of other DNA damage-related drugs across different risk groups. Molecular assays were performed to investigate the role of the key gene TONSL in breast cancer.

**Results:**

By integrating data from public database, we established a prognostic signature comprising thirteen DDR genes. Our analysis indicated that this model is associated with immune infiltration patterns in breast cancer patients, particularly concerning CD8+ T cells and NK cells. Additionally, it demonstrated a significant correlation with sensitivity to other DDR-related drugs, suggesting its potential as a biomarker for treatment efficacy. Compared to the control group, TONSL-knockdown cell lines exhibited a diminished response to DNA-damaging agents, marked by a notable increase in DNA damage levels and enhanced drug sensitivity. Furthermore, single-cell analysis revealed elevated TONSL expression in dendritic and epithelial cells, particularly in triple-negative breast cancers.

**Conclusions:**

Carboplatin resistance-related DDR genes are associated with prognosis, immune infiltration, and drug sensitivity in breast cancer patients. TONSL may serve as a potential therapeutic target for breast cancer, particularly in triple-negative breast cancer, indicating new treatment strategies for these patients.

## 1 Introduction

Breast cancer is one of the most prevalent malignant tumors worldwide. According to cancer statistics published in early 2024 by the American Cancer Society in the journal CA Cancer J Clin (1), it remains the most frequently diagnosed cancer and the second leading cause of cancer-related deaths in women. Triple-negative breast cancer (TNBC) accounts for 15–20% of the total population (1). Compared with hormone receptor-positive and HER2-amplified tumors, TNBC is the most aggressive subtype, with higher recurrence rates, earlier mortality in operable stages (2), and shorter overall survival in patients with inoperable tumors (3, 4).

Cytotoxic chemotherapy remains the most common systemic treatment options for TNBC patients (5). It mainly includes protein synthesis inhibitors like paclitaxel; DNA synthesis inhibitors such as alkylating agents (e.g., cyclophosphamide), platinum-based compounds (e.g., cisplatin and carboplatin), and topoisomerase inhibitors; as well as RNA synthesis inhibitors, like anthracyclines (e.g., doxorubicin). Carboplatin, alone or in combination with other drugs, is a first-line chemotherapeutic agent for breast cancer, particularly TNBC (6). Clinical trials have demonstrated its therapeutic benefits in TNBC. The BrighTNess trial showed significantly higher pCR rates in carboplatin-containing groups (53% and 58%, respectively) compared to paclitaxel alone (31%) during neoadjuvant treatment (7). Consistent benefits of carboplatin combination therapy were observed in two other clinical trials (8, 9). Carboplatin induces apoptosis in tumor cells by disrupting normal DNA function through intrastrand and interstrand cross-linking (10). Despite its efficacy, resistance to carboplatin remains a significant challenge. Many TNBC patients develop resistance to carboplatin, leading to incomplete eradication of lesions or even progression of the disease due to uncontrolled tumor growth. This resistance contributes to a poorer prognosis and lower survival rates, particularly within the first three years post-treatment (11, 12). Moreover, the complexity and heterogeneity of breast cancer also lead to varying responses to carboplatin (6). However, the specific mechanisms underlying carboplatin resistance in TNBC remain largely unknown.

Previous studies indicated that variations in DNA damage repair (DDR) significantly influence drug sensitivity. During cancer evolution, most cancers lose their key DDR pathways (13, 14). While normal cells can still respond to damage appropriately, tumor cells with defective or enhanced DDR mechanisms often evade complete eradication, leading to adverse events such as recurrence and metastasis. For example, glioma stem cells exhibit elevated DNA damage response, making them resistant to radiotherapy (15). As a result, researchers hypothesize that inhibiting the DDR could improve the effectiveness of both radiotherapy and chemotherapy. Additionally, the DDR influences tumor immunogenicity, including tumor cell-autonomous responses and tumor cell-microenvironment interactions (16, 17). DDR deficiency can stimulate the innate immune system or enhance adaptive immune recognition of tumors by increasing the activation of the STING pathway (18). However, how DDR-related genes impact immune infiltration and drug sensitivity in breast cancer remains unclear.

In recent years, 3D culture model has been increasingly utilized in anti-tumor drug research. This model overcomes the limitations of traditional 2D *in vitro* cultures, offering more valuable insights into cell-cell and cell-matrix interactions, as well as heterogeneity and structural complexity. It retains more physiologically relevant states of tumors *in vivo* and provides a more clinically representative response to therapeutic drugs (19). Comparative studies of gene and protein expression indicate that levels of metabolism, cellular stress response, structure, signal transduction, and cellular transport proteins are higher in spheroids than in 2D cultured cells (20). Moreover, it allows for the evaluation of the tumor microenvironment’s (TME) impact on tumors, bridging the gap between 2D culture models and the *in vivo* ecosystem.

In this study, we constructed a DDR gene-based classification model using transcriptomic data from the 3D carboplatin-resistant model (CBRD) to explore its potential prognostic value. According to the risk score, we divided breast cancer patients into high- and low-risk groups and compared their prognosis, biological characteristics, pathway enrichment, and impact on immune infiltration and drug sensitivity. Specifically, we further explored this model in TNBC. Finally, we conducted single-cell analysis and functional studies on key genes identified within the model.

## 2 Methods and materials

### 2.1 2D cell culture

Immortalized human triple-negative breast cancer cell lines MDA-MB-231 and 293T cells was obtained from the American Type Culture Collection (ATCC; Manassas, VA, USA). The cells were cultured in complete DMEM (BasalMedia, Shanghai, China) containing 10% Fetal Bovine Serum (Lonsa Science SRL, Uruguay) at 37°C with 5%CO_2_.

### 2.2 3D cell culture

The 3D on-top culture model was constructed as previously described (21–23). Briefly, all the necessary materials were prechilled one day in advance. The BME gel needed to be thawed at 4°C one day in advance. The surface of the prechilled 6-well plate was coated with a thin layer of 500ul BME (Cultrex UltiMatrix Reduced Growth Factor Basement Membrane Extract, R&D Systems, MN, USA) evenly with prechilled pipette tip and incubate for 15-30 min at 37°C to allow the BME to gel. The cells were trypsinized from a monolayer to a single-cell suspension. Then pelleted the cells by centrifugation at ~115 g and resuspended cells in half the “medium volume” (1ml) and plate onto the coated surface. The cells were allowed to settle and attach to the BME gel for 10-30 min at 37°C. The remaining medium (1ml) was chilled on ice and add BME to 10% volume then added the BME-medium mixture to the plated culture. The culture was maintained with or without indicated drugs. BME-medium mixture was replaced every 2 days. The 3D spheroids were treated with organoid harvest solution to dissolve the Matrigel, followed by centrifugation to remove the gel, yielding cell spheroids.

### 2.3 Transcriptome sequencing

Total mRNA was extracted via TRIzol (Invitrogen, USA). RNA integrity was assessed via the RNA Nano 6000 Assay Kit of the Bioanalyzer 2100 system (Agilent Technologies, CA, USA). Total RNA was used as input material for the RNA sample preparations. Briefly, mRNA was purified from total RNA via poly-T oligo-attached magnetic beads. Fragmentation was carried out using divalent cations under elevated temperature in First Strand Synthesis Reaction Buffer (5X). First-strand cDNA was synthesized via random hexamer primers and M-MuLV reverse transcriptase (RNase H-). Second-strand cDNA synthesis was subsequently performed via DNA polymerase I and RNase H. The remaining overhangs were converted into blunt ends via exonuclease/polymerase activities. After adenylation of the 3’ ends of the DNA fragments, adaptors with hairpin loop structures were ligated to prepare for hybridization. To preferentially select cDNA fragments 370~420 bp in length, the library fragments were purified with the AMPure XP system. Then, PCR was performed with Phusion High-Fidelity DNA polymerase, universal PCR primers and Index (X) primers. Finally, the PCR products were purified (AMPure XP system), and library quality was assessed on an Agilent Bioanalyzer 2100 system.

### 2.4 Construction of a risk score system based on DDR genes

To begin with, the DDR genes related to carboplatin-resistance were analyzed by the R package ‘DESeq2’. We compared the differentially expressed genes from the transcriptomic data of the 3D culture model. These genes were then intersected with the DDR-related gene list compiled by Alyssa L Smith et al. (24) to obtain the carboplatin-resistance related DDR genes, as illustrated in the Venn diagram. The prognostic relevance of these genes was evaluated in breast cancer patients from the TCGA database by univariate Cox regression analysis. Genes with significant impact (p<0.05) on prognosis passed through the LASSO regression for variable selection and shrinkage. The analysis generated the crucial genes participating in model construction and their corresponding coefficients by the “glmnet” R package (25). Finally, the CBRD score was calculated using the formula below, where Coefi is the coefficient and Expi is the expression value of each crucial gene.


$$CBRD\;score=\sum_{i=1}^{n}Coef_{i}\times Exp_{i}$$

### 2.5 Plasmid and shRNA transfection

The shRNA sequences used were obtained from Zorinbio (Shanghai, China). When 293T cells in the 6-well plate reached 80–90% confluency, the target plasmid containing shRNA, tool plasmid DR8.9 and VSVG were mixed with Opti-MEM. Lipofectamine 3000 (Life Technologies-Invitrogen, USA) was added to the 293T cell culture medium. After 48 h, the viral supernatant from 293T cells was collected and added to the MDA-MB-231 cell culture medium, supplemented with 10 µg/ml polybrene. Forty-eight hours later, MDA-MB-231 cells with (virus group) or without virus (control group) were treated with 2 µg/ml puromycin. When the control group cells were all killed, the surviving cells in the virus group were considered to be successfully constructed.

### 2.6 Expression level of mRNA by RT−qPCR

mRNA was isolated from 6-well plate cells with 90~100% confluency. cDNA was reversely transcribed by RT SuperMix kit (Vazyme, Nanjing, China). RT−qPCR was performed using SYBR Green Master Mix (Vazyme, Nanjing, China). The data were analyzed via the △△CT method, with GAPDH used as a housekeeping gene. The primers used were as follows: TONSL forward, 5’-CCGCCTCTATCTCAACCTGG-3’; TONSL reverse, 5’-AGGTCCTCGTAAAGGTGGTTC-3’.

### 2.7 Viability measurements

Three thousand cells were seeded into a 96-well plate. After 24 h, the cells were treated with the indicated doses of drugs for 72 h. A Cell Counting Kit-8 (Dojindo, Japan) was used to assess cell viability. The absorbance at a wavelength of 450 nm was measured for each well. The half-maximal inhibitory concentration (IC50) was used to assess the sensitivity of the cells to the drugs.

### 2.8 Comet assay

A comet assay was performed as previously described (26). Briefly, the cells were treated as indicated, mixed gently with 0.08% low-melting point agarose, spread on glass slides prepared with a 0.08% normal melting point agarose layer, and a coverslip was added on top. Once dried, the coverslips were removed, and the slides were then submerged in precooled lysis buffer at 4°C for 60 min. After rinsing, the slides were then placed in an electrophoresis apparatus with running buffer just covered and run for 20 min at 25 V. Then, the samples were stained with nucleic acid dye and captured under an Olympus IX81 confocal microscope.

### 2.9 Immunofluorescence

Cells were seeded on 4-chamber glass bottom dishes (Cellvis, CA, USA) and incubated overnight in culture medium at 37°C with 5%CO_2_. The cells were treated with the indicated drugs at the indicated dosages. After treatment, the cells were fixed with 4% paraformaldehyde and permeabilized with 0.05% Triton X-100 in PBS at room temperature. The cells were then blocked with 5% BSA for 1 hr, incubated overnight with the indicated primary antibodies at 4°C and then exposed to secondary antibodies and DAPI. The images were observed with a ZEISS LSM880 Airyscan.

### 2.10 SDS−PAGE and western blotting

Total protein was isolated from the cells via lysis buffer containing a protease inhibitor cocktail (MCE, NJ, USA). The proteins were separated by SDS−PAGE and transferred onto PVDF membranes. After being blocked with 5% nonfat milk powder for 1 hr, the PVDF membrane was incubated with the target primary antibody overnight at 4°C, followed by secondary antibody incubation for 1 hr at room temperature. The image of the target band was visualized with a chemiluminescence kit.

### 2.11 Bioinformatics analysis

The breast cancer expression data and survival information files were downloaded from the TCGA (The Cancer Genome Atlas) database and GEO database, including GSE20685 (302 samples) and GSE86166 (355 samples), via R software. Single-cell RNA sequencing data were obtained from the EMTAB8107, GSE150660, GSE161529 and GSE176078 datasets. To ensure consistency in data analysis, the RNAseq raw read count from the TCGA database was converted to fragments per kilobase of exon model per million mapped fragments (FPKM) and subsequently log-2 transformed. The data of the GEO database were sourced from the Affymetrix^®^ GPL570 platform (Human Genome U133 Plus 2.0 Array). We reannotated the probe sets of the GPL570 array for genes by mapping all probes to the human genome (hg38).

### 2.12 Single-cell RNA sequencing analysis

Genes expressed in fewer than three cells were excluded, and cells expressing fewer than 200 genes were filtered out. A scrublet was used to remove the doublets. Further quality control was performed on the basis of the percentage of mitochondrial gene counts, filtering out cells with > 16.38% mitochondrial gene counts. We then applied library size normalization via the “scanpy.pp.normalize_total” function in Scanpy to normalize the data matrix.

Dimensionality reduction and unsupervised clustering were performed on the basis of the workflow in Scanpy. The “scanpy.pp.highly_variable_genes” function was used to select highly variable genes for downstream analysis, identifying the top 4000 highly variable genes. We then regressed out the effects of total counts and the percentage of expressed mitochondrial genes for each cell via the “scanpy.pp.regress_out” function. Additionally, we scaled each gene to unit variance via “scanpy.pp.scale” with the parameter “max_value = 10.” After data preprocessing, we reduced the dimensionality of the data via principal component analysis (PCA). To remove batch effects from different datasets, we executed batch integration with the parameters “n_pcs = 47” using “sc.external.pp.harmony_integrate.” Finally, we used UMAP implemented by the “scanpy.tl.umap” function to further reduce the dimensionality of the merged dataset, followed by clustering the cell neighborhood graph via the Leiden clustering method.

### 2.13 Xenograft mouse model

A total of 5×106 MDA-MB-231 shNC or shTONSL cells were injected subcutaneously into the flanks of 4–6-week-old female nude mice. The tumor volume and mouse weight were observed and measured every two to three days. Once the tumor volume reached approximately 50 mm3, carboplatin at a dosage of 100 mg/kg was administered via intraperitoneal injection once a week until sacrifice.

### 2.14 Assessment of immune infiltration

The immune-related signature gene sets were used to compare the differences in the expression of immune-related markers between the two groups. Various immune infiltration scoring algorithms, including TIMER, CIBERSORT, QUANTISEQ, MCPCOUNTER, XCELL, and EPIC, were applied. Additionally, the ESTIMATE score and ssGSEA were used to further assess tumor purity.

### 2.15 Prediction of drug sensitivity

The evaluation of differences in drug susceptibility to DNA damage or genome stability-targeting drugs among patients with different risk scores was primarily conducted via the Genomics of Cancer Drug Sensitivity (GDSC) database (27) and machine learning models for prediction. The R package ‘oncoPredict’ (28) was used to calculate the estimated IC50 values and determine significant differences. Compounds from the GDSC1 and GDSC2 databases mainly targeting DNA damage and genomic stability were selected for drug sensitivity analysis (**Supplementary Table S2**). The expression data and drug response data of cell lines from GDSC1 and GDSC2 were used as training datasets, respectively. The estimated IC50 of compounds for the target patients was calculated through the ‘calcPhenotype’ function. Subsequently, the patients were divided into two risk groups based on the CBRD score, and statistical tests were performed on the estimated IC50 between the two groups.

### 2.16 Statistical analysis

All expression data analysis and statistical analyses were performed via R and R Studio software (R 4.3.3). Student’s t test or the Wilcoxon test was used to compare significant differences between groups. Univariate Cox regression models and Kaplan−Meier curves were used to analyze survival. A two-tailed p value test was conducted, with statistical significance defined as p < 0.05.

## 3 Results

### 3.1 Establishment of a 3D culture model of the MDA-MB-231 cell line and drug treatment

**Figure 1:**
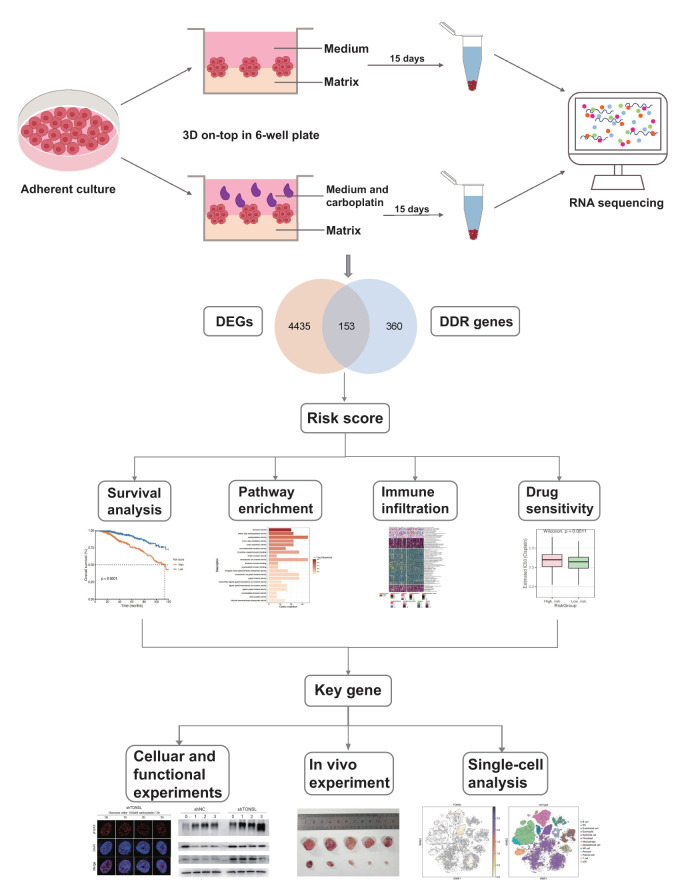
Flow chart of the study. Carboplatin-resistant and -sensitive models were established by 3D culture system and performed transcriptome sequencing, identifying 153 DDR genes associated with carboplatin resistance. By integrating these findings with TCGA data, we conducted univariate regression analysis to identify candidate genes associated with prognosis. Subsequently, we applied a LASSO regression model to construct a 13-gene risk score system. This system was further analyzed for pathway enrichment, immune infiltration, and drug sensitivity between high- and low-risk groups. Within this model, we selected TONSL as a key gene and conducted molecular experiments to investigate its roles in DNA damage repair and immune infiltration.

**Figure 2:**
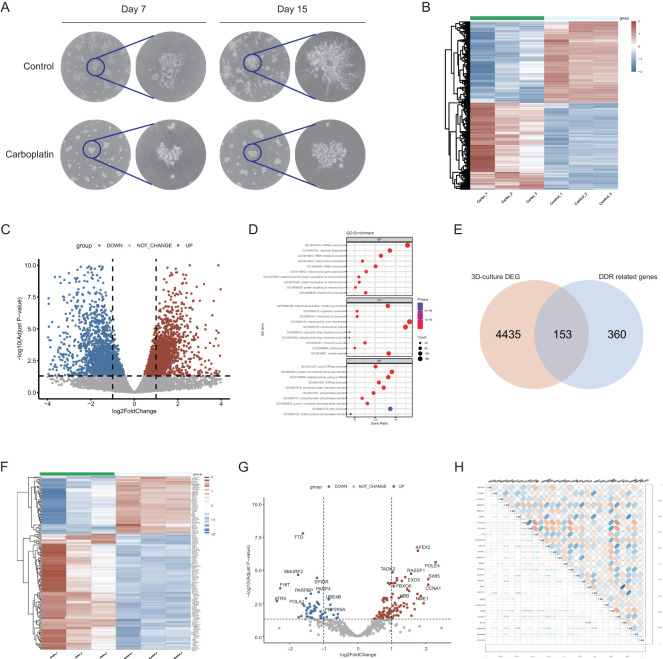
Establishment of 3D-culture model and identification of carboplatin resistance-related DDR genes. **(A)** The morphology of the 3D culture model on days 7 and 15 with Overview image and zoomed-in image. **(B)** Heatmap of expression profiles of 3D-culture model comparing carboplatin-treated group to control group. Red blocks represent genes with upregulated expression. Blue blocks represent genes with downregulated expression. **(C)** Volcano plot of expression profiles of 3D-culture model comparing carboplatin-treated group to control group. **(D)** GO enrichment analysis of the differentially expressed genes of 3D-culture model. The top 10 enriched pathways are displayed for each category. **(E)** The intersecting genes between the DEGs of 3D culture model and DDR gene list. **(F)** Heatmap of expression profile of 153 overlapped DDR genes in carboplatin-treated group compared to control group. Red blocks represent genes with upregulated expression. Blue blocks represent genes with downregulated expression. **(G)** Volcano plot of expression profile of 153 overlapped DDR genes. **(H)** Correlation heatmap among the 27 DDR genes associated with prognosis and their correlation coefficient.

3D culture is increasingly favored for exploring gene functions and signaling pathways (29). It captures various aspects of cancer biology by mimicking the *in vivo* tumor structure (30). Dhimolea et al. (31) used a 3D culture model to simulate drug-persistent residual tumors. **Figure 1** illustrates a visual flowchart of the study design with. We cultured the MDA-MB-231 cell line under 3D conditions. After sphere formation, 100 µM carboplatin was added to the medium, and the cells were continuously cultured for 15 days (**Figure 2A**). The gel was then dissolved, and the surviving cells were collected for RNA sequencing. The control group was defined as carboplatin-sensitive group, while the surviving cells from the carboplatin-treated group were considered carboplatin-resistant. Compared to the carboplatin-treated group, the control group exhibited larger and more irregularly shaped spheres.

### 3.2 Identification of carboplatin resistance-related DDR genes associated with clinical outcomes in patients with breast cancer

To investigate the DDR genes associated with carboplatin resistance and their impact on prognosis of breast cancer, we first obtained differentially expressed genes (DEGs) by comparing the carboplatin-resistant group to the sensitive group from the transcriptomic data of 3D culture model. Genes upregulated in the resistant group are highlighted as red blocks and dots (**Figures 2B, C**). Enrichment analysis revealed that DEGs were mainly associated with ncRNA processing, ribosome biogenesis and protein serine/threonine kinase activity (**Figure 2D**). By intersecting the previously reported DDR gene set (23) with the above DEGs, we identified 153 DDR-related genes linked to carboplatin resistance (**Figure 2E**). Further analysis showed 102 upregulated and 51 downregulated genes among these 153 DDR-related genes (**Figures 2F, G**). Subsequently, with the clinical information from TCGA, univariate Cox regression analysis identified 27 candidate prognostic genes significantly associated with overall survival (OS) (p<0.05) (**Supplementary Table S1**). The correlations heat map with correlation coefficient (**Figure 2H**) showed no strong association among the candidate genes.

### 3.3 Construction of the prognostic model of carboplatin resistance-related DDR genes

**Figure 3:**
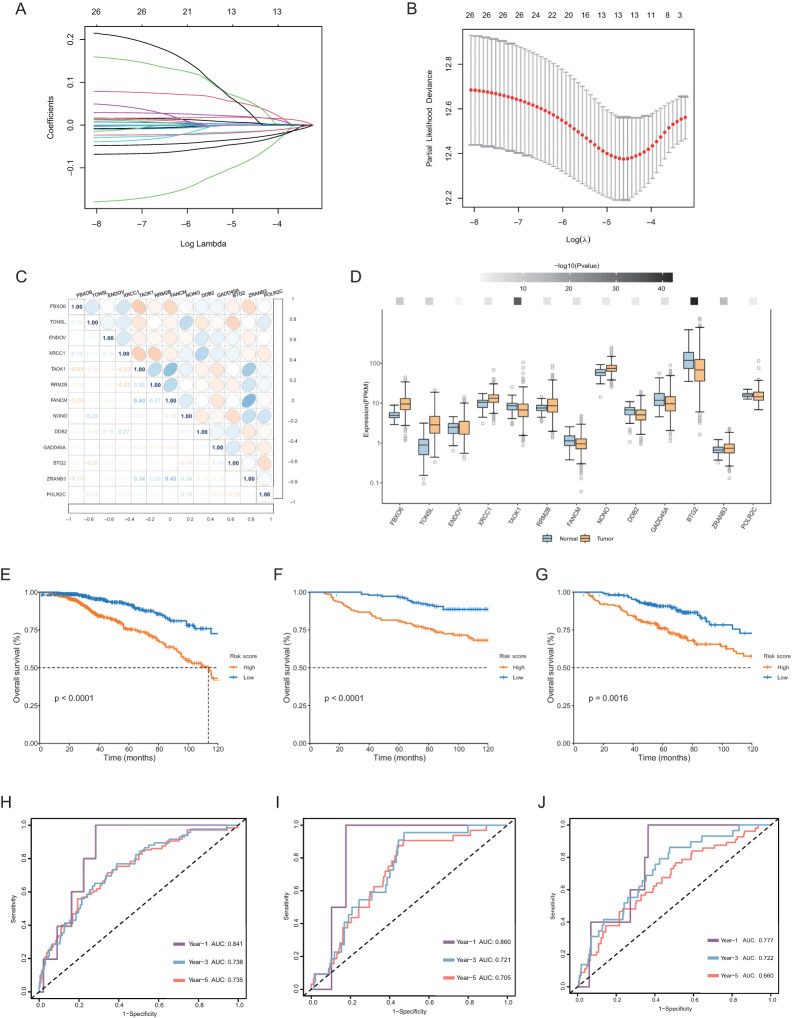
Construction of the prognostic model of carboplatin resistance-related DDR genes. **(A, B)** Charts to show the Log Lambda value and the 13 prognostic DDR genes with non-zero coefficient. **(C)** Correlation heatmap among the 13 selected genes of the risk score system and their correlation coefficient. **(D)** Differential expression of 13 genes of the risk score system in normal and tumor tissues in breast. The darker the color of the upper squares, the smaller the p-value. **(E–G)** Kaplan-Meier curve for OS in the TCGA database, GSE26085, GSE86166 datasets based on the risk score derived from the gene signature. The orange short lines represent the high-risk group, while the blue short lines represent the low-risk group. **(H–J)** ROC curve for OS in three datasets of 1, 3, 5-year.

We further included these 27 genes in a least absolute shrinkage and selection operator (LASSO) regression model. Based on the optimal value of λ (**Figures 3A, B**), we identified 13 genes through the ‘glmnet’ R package to construct a prognostic model. The correlations among those genes (**Figure 3C**) and different expression levels in tumors compared with normal tissue were determined (**Figure 3D**). All genes except ENDOV showed significant differential expression. Among them, 7 genes (TONSL, TAOK1, RRM2B, FANCM, NONO, ZRANB3 and POLR2C) had coefficients greater than 0, while 6 genes (FBXO6, ENDOV, XRCC1, DDB2, GADD45A and BTG2) with coefficients less than 0. Through this 13-gene model and formula described earlier, we calculated a risk score for each patient and used the median value as the cutoff point to divide patients into two groups. The Kaplan−Meier curve indicated that the risk score was significantly associated with OS, with high-risk patients tending to have significantly worse OS (**Figure 3E**). The areas under the curve (AUCs) for the 1-, 3-, and 5-year receptor operating characteristic (ROC) curves were 0.841, 0.738, and 0.735, respectively (**Figure 3H**).

To evaluate the performance of this risk score, we included two datasets, GSE26085 and GSE86166, for validation. Consistent with the above results, the OS rate of the high-risk group was significantly lower than that of the low-risk group (**Figures 3F, G, I, J**).

### 3.4 Biological processes and pathway activation states

**Figure 4:**
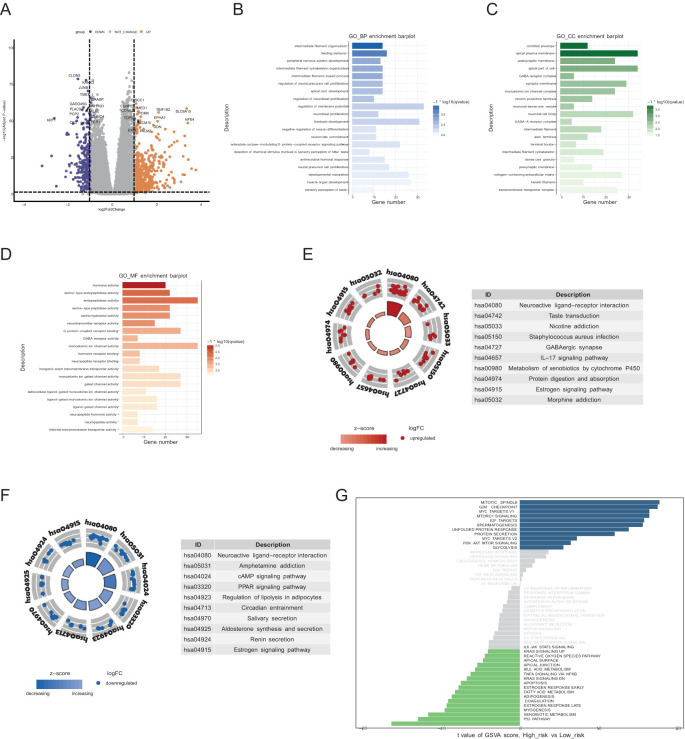
Biological processes and pathway activation states. **(A)** Volcano plot of DEGs comparing the high-risk group to the low-risk group. **(B-D)** GO terms of the BP, CC, and MF categories enriched in the DEGs. The top 20 enriched pathways are displayed for each category. **(E, F)** KEGG analysis of the two risk groups. The top 10 enriched pathways are displayed for each category in upregulated DEGs **(E)** and downregulated DEGs **(F)**. **(G)** GSVA of the two risk groups. The blue blocks represent pathways significantly upregulated in the high-risk group, while the green blocks represent pathways significantly upregulated in the low-risk group.

To explore the biological significance of the two groups, we first analyzed the DEGs between the groups comparing high risk group to low-risk group (**Figure 4A**). Genes such as SLC6A15, NFE4, and EPHA7 were significantly upregulated in the patients with high risk. GO enrichment analysis revealed that the DEGs were involved primarily in intermediate filament organization and feeding behavior in the biological process category (**Figure 4B**), cornified envelope and apical plasma membrane in the cellular component category (**Figure 4C**), and hormone activity and serine-type endopeptidase activity in the molecular function category (**Figure 4D**). KEGG pathway analysis revealed that, compared with those in the low-risk group, the upregulated DEGs in the high-risk group were enriched mainly in neuroactive ligand−receptor interactions, metabolism of xenobiotics by cytochrome P450, and the estrogen signaling pathway (**Figure 4E**); the downregulated genes were enriched primarily in the cAMP signaling pathway, the PPAR signaling pathway, and regulation of lipolysis in adipocytes (**Figure 4F**). To further explore differences between the two groups, we performed GSVA. Pathways such as the mitotic spindle, G2M checkpoint, and Myc targets were significantly upregulated in the high-risk group, whereas pathways such as the p53 pathway, xenobiotic metabolism, and myogenesis were significantly upregulated in the low-risk group (**Figure 4G**).

### 3.5 The immune landscape of the high-risk and low-risk groups

**Figure 5:**
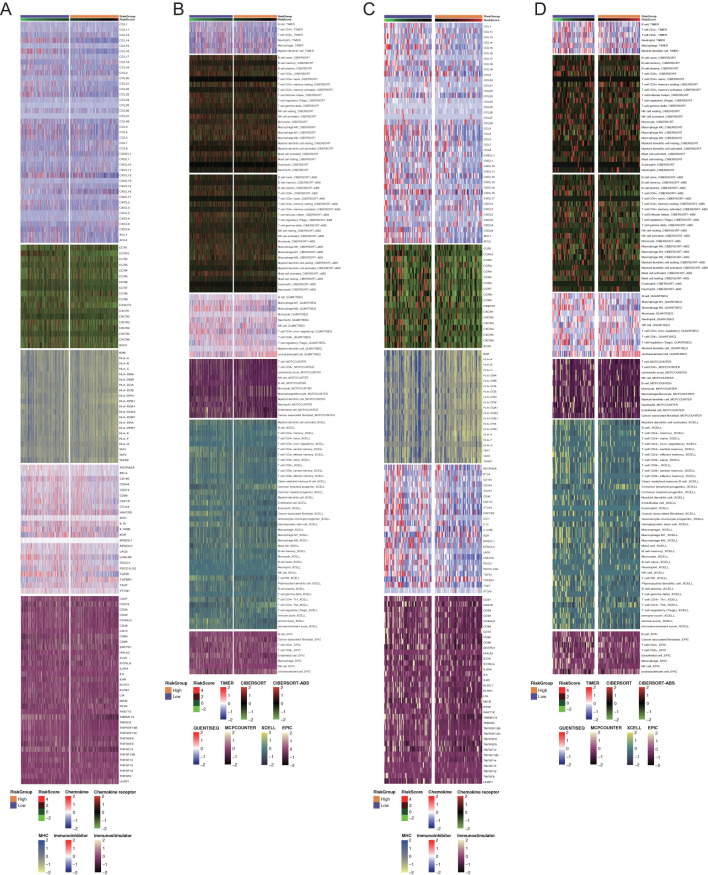
Immune profiling of the two groups in all breast cancers and triple-negative breast cancers. **(A, C)** Heatmaps of differential immune-related genes among the overall patients and TNBC patients among two risk groups. Each section with different color represented different set of genes. **(B, D)** Heatmaps of tumor-related infiltrating immune cells based on TIMER, CIBERSORT, CIBERSORT-ABS, MCPCOUNTER, QUANTISEQ, EPIC, and XCELL algorithms among the overall patients and TNBC patients among two risk groups. Each section with different color represented different algorithms.

Owing to the close interaction between the DDR and the immune system in tumor cells, we analyzed immune infiltration differences among patients classified by this DDR gene-related risk score. First, we examined five immune-related gene sets—chemokines, chemokine receptors, MHC, immunoinhibitors, and immunostimulators. In the overall cohort, genes such as CCL14, CCL16, CCR10, TGFB1, TMEM173, and TNFSF13 were significantly upregulated in the low-risk group (**Figure 5A**). Similarly, in the TNBC cohort, the immune signature genes were consistently overexpressed in the low-risk group compared with the high-risk group (**Figure 5C**). Additionally, we used several algorithms such as TIMER, CIBERSORT, CIBERSORT-ABS, MCPCOUNTER, QUANTISEQ, EPIC, and XCELL to compare immune cell composition and infiltration levels between the two groups (**Figures 5B, D**).

**Figure 6:**
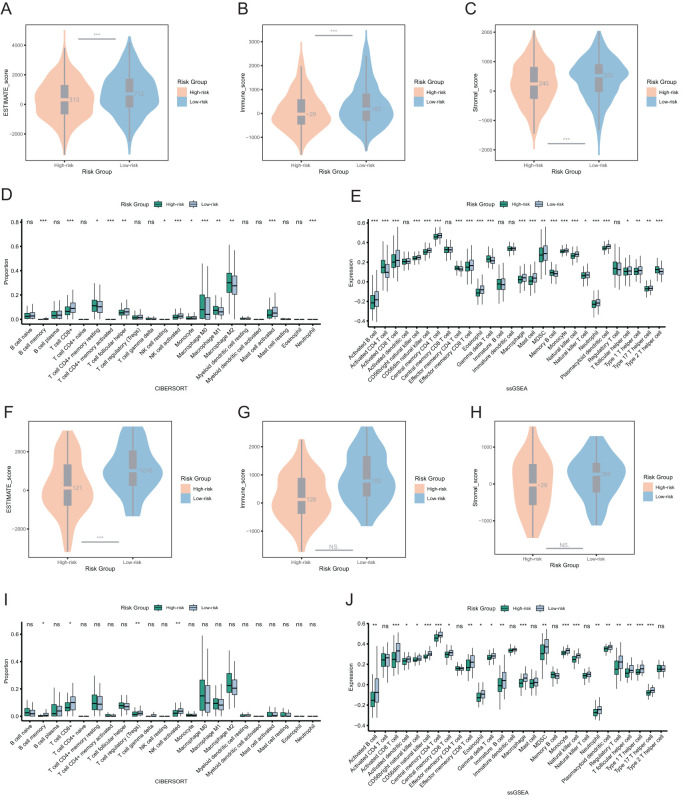
Immune landscapes and tumor purity in two risk groups. **(A)** Comparison of ESTIMATE score between two risk groups among the overall patients. **(B)** Comparison of immune score between two risk groups among the overall patients. **(C)** Comparison of stromal score between two risk groups among the overall patients. **(D, E)** The immune cell proportions between two risk groups among the overall patients through CIBERSORT and ssGSEA analysis. **(F)** Comparison of ESTIMATE score between two risk groups among the TNBC patients. **(G)** Comparison of immune score between two risk groups among the TNBC patients. **(H)** Comparison of stromal score between two risk groups among the TNBC patients. **(I, J)** The immune cell proportions between two risk groups among the TNBC patients through CIBERSORT and ssGSEA analysis. ns *p*>0.05, * *p*<0.05, ** *p*<0.01, *** *p*<0.001.

Next, we evaluated tumor purity across risk groups. In both the overall cohort and the TNBC cohort, the ESTIMATE score and immune score were significantly lower in the high-risk group, indicating reduced immune infiltration and a higher proportion of tumor cells in the tumor microenvironment of the high-risk group (**Figures 6A–C, F–H**). In the overall cohort, the low-risk group showed higher infiltration of CD8+ T cells, activated NK cells, mast cells, and follicular helper T cells (**Figures 6D, E**). Similarly, in the TNBC cohort, overall immune cell infiltration was greater in the low-risk group, particularly for CD8+ T cells, regulatory T cells (Tregs), and activated NK cells (**Figures 6I, J**).

These findings suggested that breast cancer patients with high-risk scores have lower immune cell infiltration. Conversely, patients with low-risk scores, especially those with TNBC, may be more sensitive to immunotherapy.

### 3.6 Analysis of drug sensitivity in high- and low-risk groups

**Figure 7:**
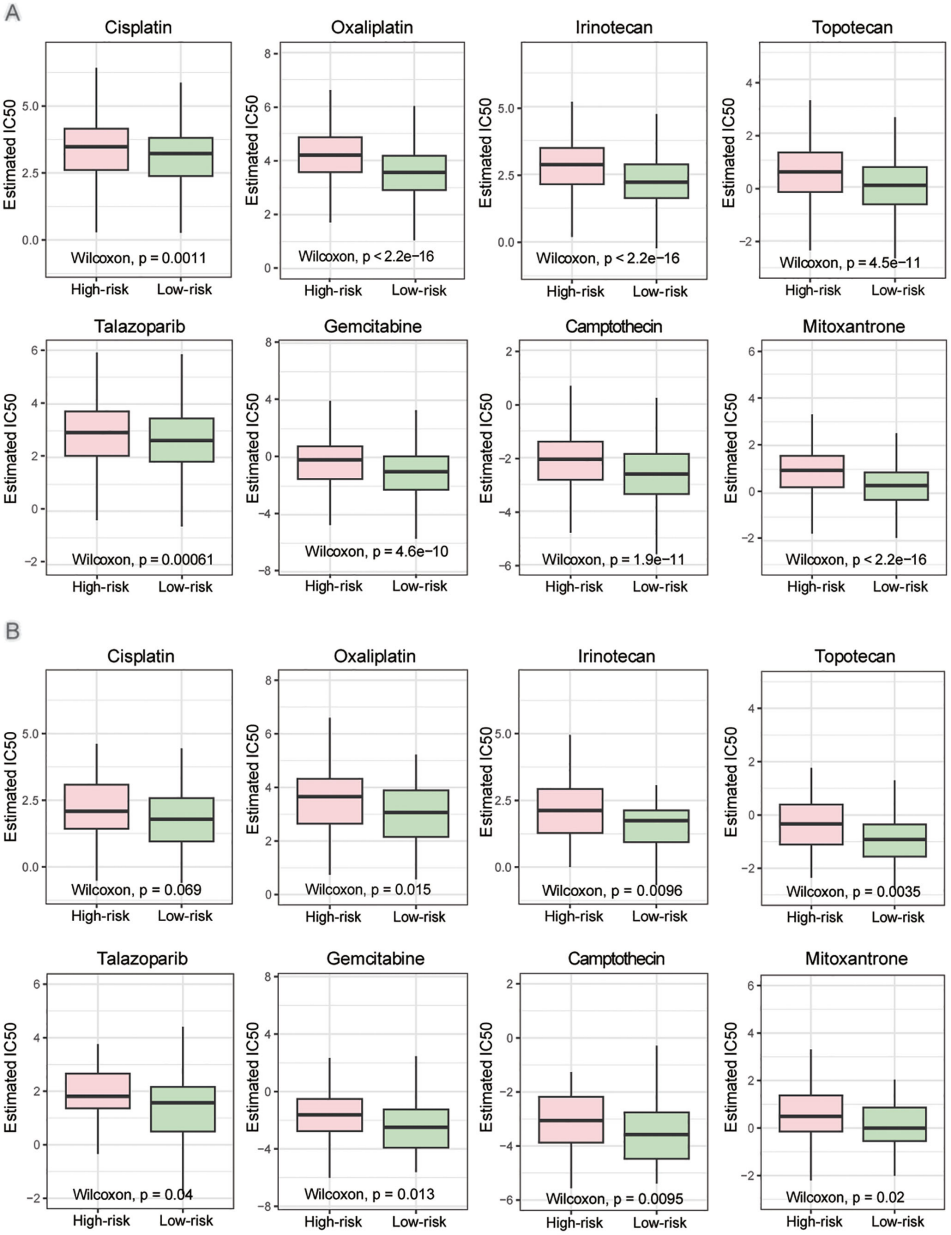
Drug sensitivity in high- and low-risk groups. **(A)** Estimated IC50 of the indicated drugs among two risk groups in the overall patients. **(B)** Estimated IC50 of the indicated drugs among two risk groups in the TNBC patients. The statistical test was the ‘Wilcoxon test’.

We further investigated the therapeutic responses of patients in two risk groups to other DNA damage drugs and genome stability-related drugs. In both overall breast cancer and TNBC patients, the high-risk group exhibited significantly lower drug sensitivity compared to the low-risk group (**Figure 7** and **Supplementary Figure S1**). Specifically, notable differences were observed in sensitivity to the platinum drug oxaliplatin, the topoisomerase inhibitors irinotecan and topotecan, the PARP inhibitor talazoparib, and the cytarabine derivative gemcitabine. These differences were statistically significant among all breast cancer patients (**Figure 7A**) and TNBC patients (**Figure 7B**). The findings were validated using the independent dataset GSE20685 (**Supplementary Table S3**), which confirmed that the high-risk group defined by this risk score model has reduced sensitivity to DNA damage-related drugs. This risk score may serve as a valuable tool for guiding drug selection in breast cancer treatment.

### 3.7 The role of the hub gene TONSL

**Figure 8:**
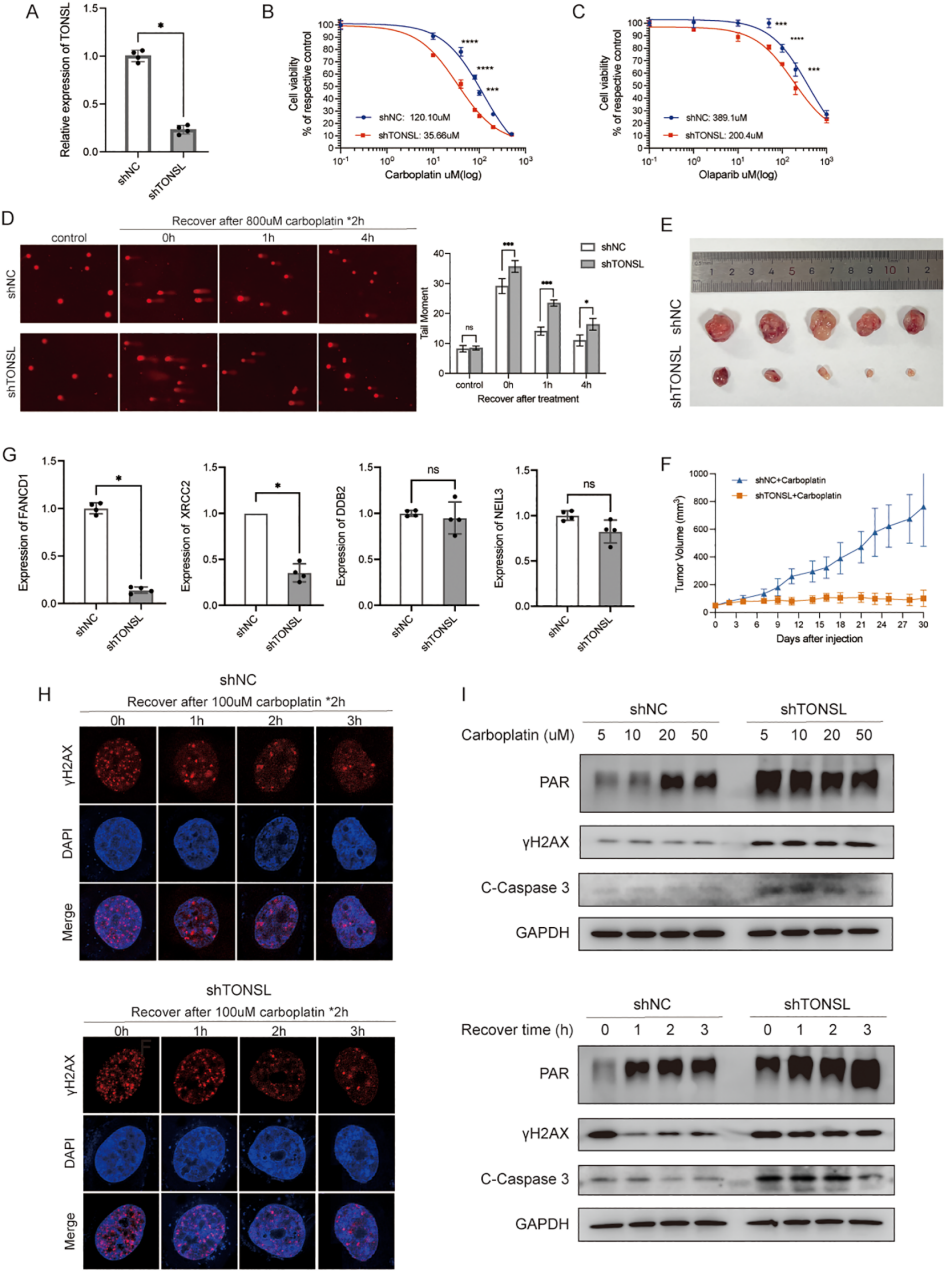
The role of gene TONSL in DNA damage response. **(A)** RT-qPCR of knockdown efficiency of TONSL in MDA-MB-231 cells. **(B, C)** The cell viability of MDA-MB-231 reciving carboplatin and olaparib with or without TONSL knockdown. **(D)** Comet assay of shNC cells with 0h, 1h, 4h recovery time after treatment compared with shTONSL cells and quantitative analysis of tail moment. **(E)** Subcutaneous transplantation tumor isolates. **(F)** Tumor growth of shNC/shTONSL MDA-MB-231 cells in vivo with injection of carboplatin. **(G)** RT-qPCR of FANCD1, XRCC2, DDB2 and NEIL3 between shNC/shTONSL MDA-MB-231 cells. **(H)** Immunofluorescence staining of γH2AX with different recovery time after carboplatin treatment in shNC and shTONSL cells. **(I)** Western blot of cells treated with different doses of carboplatin for two hours or recovered for different time after same treatment of carboplatin. ns *p*>0.05, * *p*<0.05, *** *p*<0.001, **** *p*<0.0001.

To further understand the role of these DDR genes in cellular regulation, we selected TONSL for exploration. TONSL exhibited the highest hazard ratios among the model genes significantly overexpressed in tumor tissues and was associated with OS in breast cancer patients (**Figure 3B**, **Supplementary Table S1**). We then successfully knocked down TONSL in MDA-MB-231 cells via shRNA (**Figure 8A**). CCK8 assays showed that TONSL knockdown significantly increase cell sensitivity to carboplatin (**Figure 8B**) and olaparib (**Figure 8C**) compared to control cells. Comet assay revealed reduced DNA repair efficiency in TONSL-knockdown cells following carboplatin treatment (**Figure 8D**). In the mouse xenograft tumor models, tumors with reduced TONSL expression showed an improved response to carboplatin, resulting in smaller tumor sizes compared to the control group (**Figures 8E, F**). To investigate the potential regulatory role of TONSL in DNA damage repair, we referenced studies (32, 33) and validated key gene expressions via RT-PCR. TONSL knockdown significantly reduced the expression of FANCD1 and XRCC2, both key players in the Fanconi anemia and homologous recombination repair pathways, while DDB2 and NEIL3 showed no statistically changes (**Figure 8G**). Immunofluorescence staining indicated an increase in nuclear γH2AX foci, a DNA damage marker, in TONSL-knockdown cells (**Figure 8H**). Additionally, these cells exhibited elevated PAR chain production, γH2AX levels, and cleaved caspase-3 expression following treatment (**Figure 8I**). These results collectively suggest that TONSL enhances DNA repair by regulating FANCD1 and XRCC2 expression and that its reduction sensitizes breast cancer cells to carboplatin.

**Figure 9:**
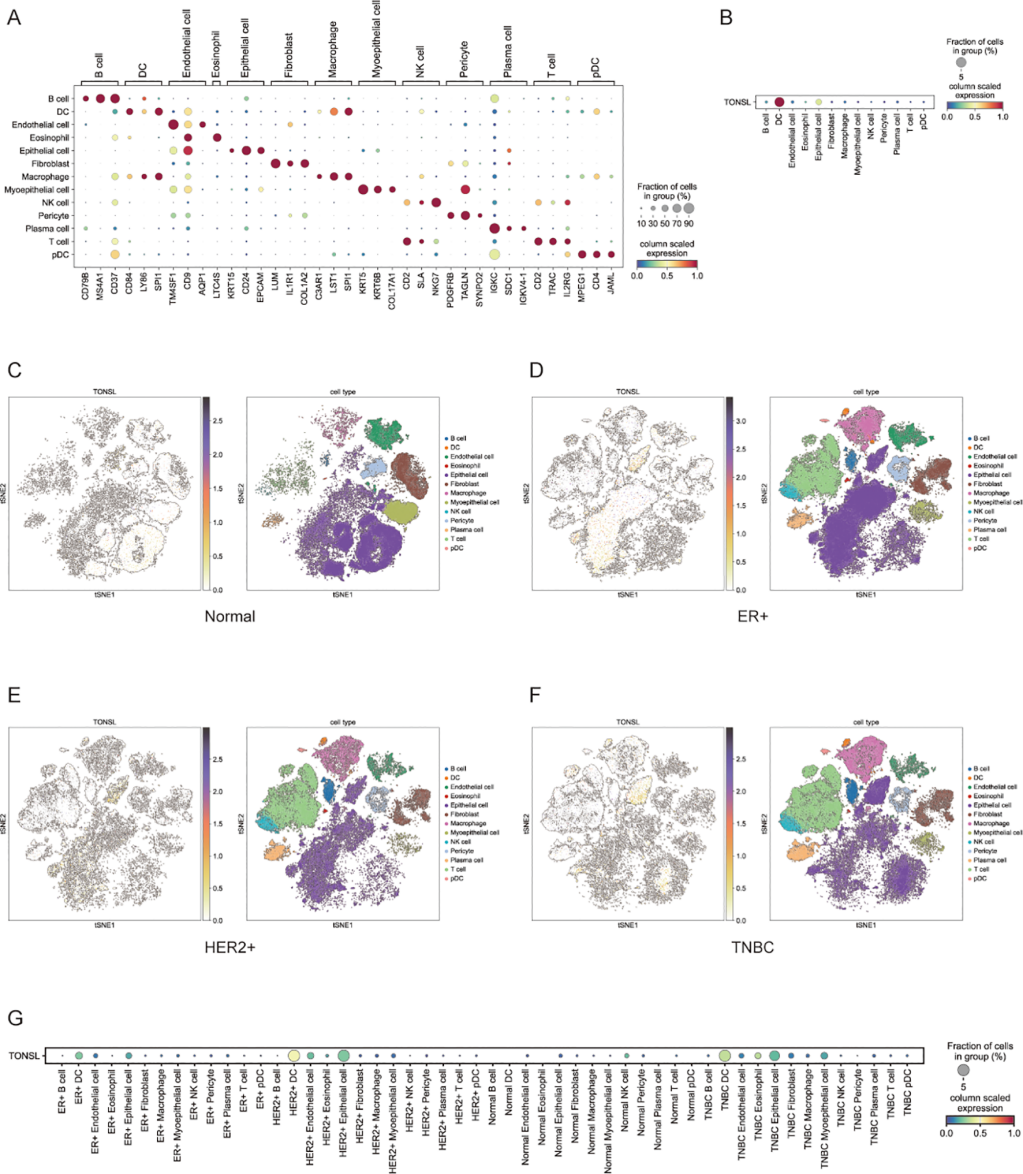
Expression of TONSL in different cell types. **(A)** Major cell types based on characteristic gene expression. **(B)** Expression of TONSL in 13 cell types. **(C-F)** tSNE plot of intratumoral immune cells and TONSL showing the correlation between infiltration of different immune cells and TONSL expression across normal tissue, ER+ breast cancer, HER2+ breast cancer and TNBC. **(G)** Dot plot of expression of TONSL through major cell types in tissues among normal and different molecular subtype.

We also performed single-cell analysis to investigate TONSL expression across different cell types. Thirteen major cell types were identified on the basis of characteristic gene expression (**Figure 9A**). Among these 13 cell types, TONSL was expressed primarily in dendritic cells (DCs) and epithelial cells (**Figure 9B**). High expression was observed in epithelial cells across normal, ER+ breast cancer, HER2+ breast cancer, and TNBC tissues (**Figures 9C–G**). Notably, TONSL was specifically overexpressed in DCs within the TNBC population (**Figure 9F**).

## 4 Discussion

As the most prevalent malignant tumor among women worldwide, breast cancer urgently requires treatment strategies that improve prognosis and enhance quality of life. Carboplatin, a DNA cross-linking agent that induces DNA strand breaks, is one of the first-line treatments for breast cancer, particularly for TNBC patients. However, resistance to carboplatin remains a significant clinical challenge, often resulting in tumor recurrence and metastasis. The development of a prognostic model holds considerable clinical relevance. By integrating key genes associated with drug resistance, prognosis, and tumor biology, the risk score system enables precise stratification of patients into high- and low-risk groups. This stratification not only aids in predicting prognosis but also identifies individuals who may benefit from tailored therapeutic interventions (34, 35). A DDR gene model associated with carboplatin resistance could serve dual purpose. On one hand, it may help predict prognosis and identify high-risk breast cancer patients with potential carboplatin resistance, who may benefit from alternative therapeutic strategies. This allows real-time monitoring of drug efficacy and timely adjustments to treatment regimens to prevent resistance and disease progression. On the other hand, it provides an assessment of prognostic risk, enabling personalized follow-up management strategies. High-risk patients, as identified by the model, would require more frequent follow-up and monitoring to prevent adverse events and improve long-term outcomes. Thus, such a model could significantly enhance personalized care in breast cancer management, particularly for patients with limited responsiveness to platinum-based and DNA-damaging agents.

In addition to conventional factors affecting drug sensitivity—such as drug accumulation, drug efflux, factors that prevent drugs from interacting with their targets, pathways that eliminate target damage, and those involved in the cellular damage response (36, 37)—the molecular mechanisms of drug resistance play a more direct role in determining clinical outcomes. These mechanisms are the primary focus of current research aimed at addressing and overcoming platinum resistance. In this study, on the basis of transcriptomic data from our 3D-culture carboplatin-resistant model, we constructed and validated a risk score model. Using this model, breast cancer patients were stratified into high- and low-risk groups. Patients in the high-risk group exhibited significantly worse prognoses compared with those in the low-risk group. We further analyzed the DEGs, enriched pathways, and biological functions between the two groups. These findings provide insights into the molecular mechanisms underlying carboplatin resistance and suggest potential avenues for therapeutic intervention.

The immune microenvironment plays multiple roles *in vivo*, influencing various physiological processes such as inflammation, tissue repair, and immune surveillance. It also regulates tumor growth and metastasis, making it a valuable therapeutic target. For instance, modulating the inflammatory immune microenvironment has been shown to promote healing in muscle injuries (38). In tumors, based on immune infiltration levels, they can be classified as immune-cold or immune-inflamed. Transforming the immune microenvironment of immune-cold tumors and enhancing immune cell activity can bolster the immune system’s ability to attack tumors, thereby improving drug response and survival outcomes (39, 40). DNA damage has been shown to trigger innate immune responses, largely due to the accumulation of nuclear DNA in the cytoplasm (41, 42). A hallmark of malignant tumors is genomic instability (43), where DNA double-strand breaks and replication stress continuously drive chromosomal instability (44). This process is closely associated with the activation of the interferon gene (STING) pathway, which is mediated by the cGAS sensor protein that stimulates cytoplasmic DNA, forming a crucial node between cancer cells and the immune microenvironment (45). Conversely, an activated innate immune system can suppress tumorigenesis by eliminating senescent cells with oncogene activation or chronic DNA damage through the production of reactive oxygen species (ROS) and reactive nitrogen species (RNS) (46, 47). Therefore, we further analyzed immune infiltration levels and drug sensitivity across different risk score populations. Compared with the high-risk group, the low-risk group exhibited higher levels of immune infiltration, especially of CD8+ T cells and NK cells, as well as greater sensitivity to DNA damage-related drugs. CD8+ T cells and NK cells are pivotal players in tumor immunity. Tumor-specific CD8+ T cells recognize and eliminate cancer cells by detecting tumor-derived peptides presented on MHC I molecules through their T-cell receptors (48). NK cells identify tumor cells via germline-encoded activating receptors, and most circulating NK cells exist in a cytotoxic effector state, enabling NK cells to mount antitumor immune responses at early stages (49). These two cell types exhibit complementary roles in tumor immune response (50). Among patients in high-risk group, reduced immune infiltration suggests potential benefits from combination immunotherapies, such as PD-1/PD-L1 inhibitors, targeted suppression of inhibitory cytokines, cancer vaccines, or autologous immune cell infusions, may enhance antitumor efficacy (50). Recent advancements have also led to the development of cancer vaccines capable of inducing dual antitumor responses from T cells and NK cells (51). Therefore, the CBRD risk score system may provide potential predictive value for combination immunotherapy strategies, particularly for high-risk breast cancer patients with diminished immune infiltration.

Among the genes identified in our constructed models, we focused particularly on TONSL, which encodes the Tonsoku-like DNA repair protein. TONSL has emerged as a crucial player in homologous recombination (HR), replication fork repair, and chromatin formation (52–55). It is amplified in approximately 20% of breast cancers (56) and plays a pivotal role in addressing replication stress and DNA double-strand break (DSB) repair, particularly within the HR pathway (53). Previous studies have highlighted TONSL’s role in promoting tumorigenesis, with its overexpression upregulates DNA repair-related genes in pathways such as HR and Fanconi anemia, conferring resistance to damage (32). Through a series of functional cell experiments, we demonstrated that TONSL knockdown increases sensitivity to carboplatin and olaparib, decreases the repair efficiency for carboplatin-induced damage, and improves therapeutic efficacy. We also performed single-cell analysis to determine the expression of TONSL in the tumor immune microenvironment. Single-cell sequencing technology has become increasingly refined, providing valuable insights into various diseases and playing a crucial role in exploring cellular heterogeneity within tumor tissues (57). Traditional bulk RNA sequencing analyzes the average transcriptome of all cells within a sample, potentially masking transcriptional differences specific to certain cell types. In contrast, single-cell sequencing enables the analysis of gene expression at the individual cell level, revealing cell-to-cell communication pathways and uncovering unique cellular states within tumors. It offers a more precise understanding of the tumor microenvironment (58). Notably, TONSL is specifically overexpressed in DCs within TNBC compared with normal tissues and other subtypes. In summary, TONSL plays a significant role in tumor resistance to DNA-damaging drugs, potentially participating in the regulation of the immune microenvironment.

Among the remaining genes, studies have shown that FBXO6 can enhance sensitivity to platinum-based drugs by inhibiting CHK1 (59) and promote sensitivity to radiotherapy by mediating ubiquitination of CD147 (60). XRCC1 is a critical DNA damage repair gene involved in various repair pathways. It mediates the recruitment of PARP1 and PARP1 to sites of DNA damage and promotes resistance to platinum-based drugs (61, 62). RRM2B is typically associated with maintaining mitochondrial DNA stability (63); however, studies suggest that RRM2B is also amplified in various tumors, impacting DNA damage repair and treatment responses. NONO negatively correlates with cisplatin reactivity by regulating STAT3 activity (64). DDB2 is identified as a protein involved in nucleotide excision repair (NER), but it is not required for repairing platinum-induced DNA damage. Thus, its high expression may enhance sensitivity to platinum-based drugs (65). GADD45A is involved in DNA repair, cell cycle arrest, and apoptosis. Sustained expression of GADD45A might increase cellular sensitivity to cisplatin (66).

Specifically, this study employed a 3D culture system to construct a resistance model combined with public database for joint analysis. Compared to previous studies that typically relied solely on public database, this research demonstrated methodological innovation. The data integrated both *in vivo* and *in vitro* information, with the 3D culture model encompassing interactions among various cell types and extracellular components (67). This approach more comprehensively reflected the physiological conditions of the tumor microenvironment, providing a more holistic perspective for the study and enhancing the translational applicability of the findings.

However, this study still has certain limitations. First, the dataset sources originate primarily from cell lines and public databases, which were not fully represent the complexity and heterogeneity of human tumors. Secondly, clinical and genomic data from patients treated with carboplatin monotherapy were lacking, which hindered our ability to directly elucidate the prognostic value of platinum-resistance-related genes and enhance the robustness of our findings. Thirdly, the method for the construction of the risk model had potential constraints, such as bias in included variables, over-compression of coefficients, selection bias of regularization parameters, and instability in variable selection.

In summary, on the basis of transcriptomic data from the constructed 3D carboplatin resistance model, we established a DDR-related risk assessment model. This model not only has predictive value for prognosis but may also reveal immune infiltration, aiding in the further assessment of patients’ sensitivity to DNA-damaging drugs and the potential benefits of immunotherapy.

## Data Availability

The datasets presented in this study can be found in online repositories. The names of the repository/repositories and accession number(s) can be found below: https://www.ncbi.nlm.nih.gov/geo/, GSE280942.

## Supplementary material

Supplementary Figure 1 Drug sensitivity in high- and low-risk groups. **(A)** Estimated IC50 of the indicated drugs among two risk groups in the overall patients. **(B)** Estimated IC50 of the indicated drugs among two risk groups in the TNBC patients. The statistical test was the ‘Wilcoxon test’.
