# Supplementary figures and images for: Carboplatin-resistance-related DNA damage repair prognostic gene signature and its association with immune infiltration in breast cancer
Source: Front Immunol. 2025 Jan 29;16:1522149. doi: 10.3389/fimmu.2025.1522149 (PMC11813922; doi:10.3389/fimmu.2025.1522149)

Figure S1. Drug sensitivity in high- and low-risk groups

A

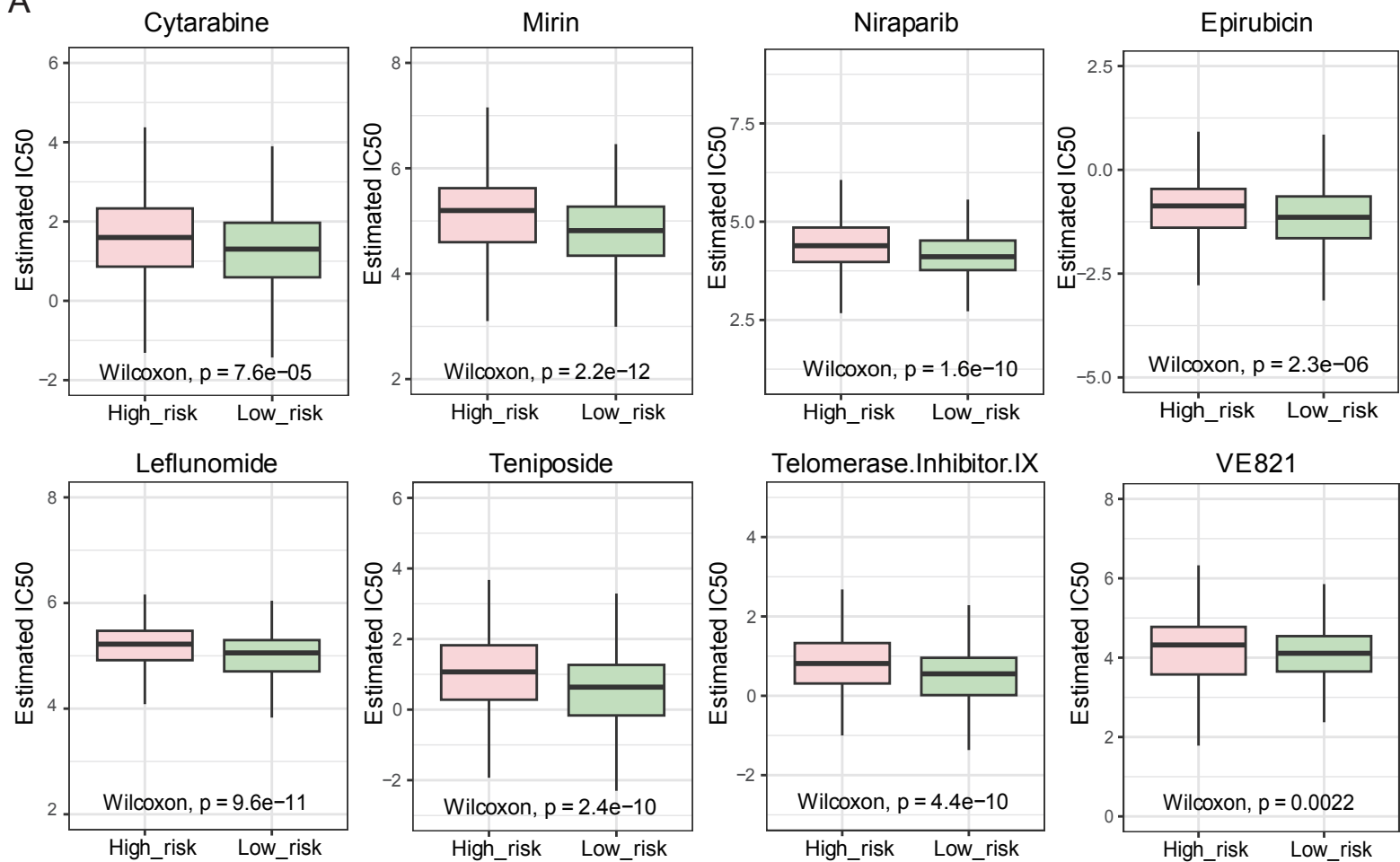

B

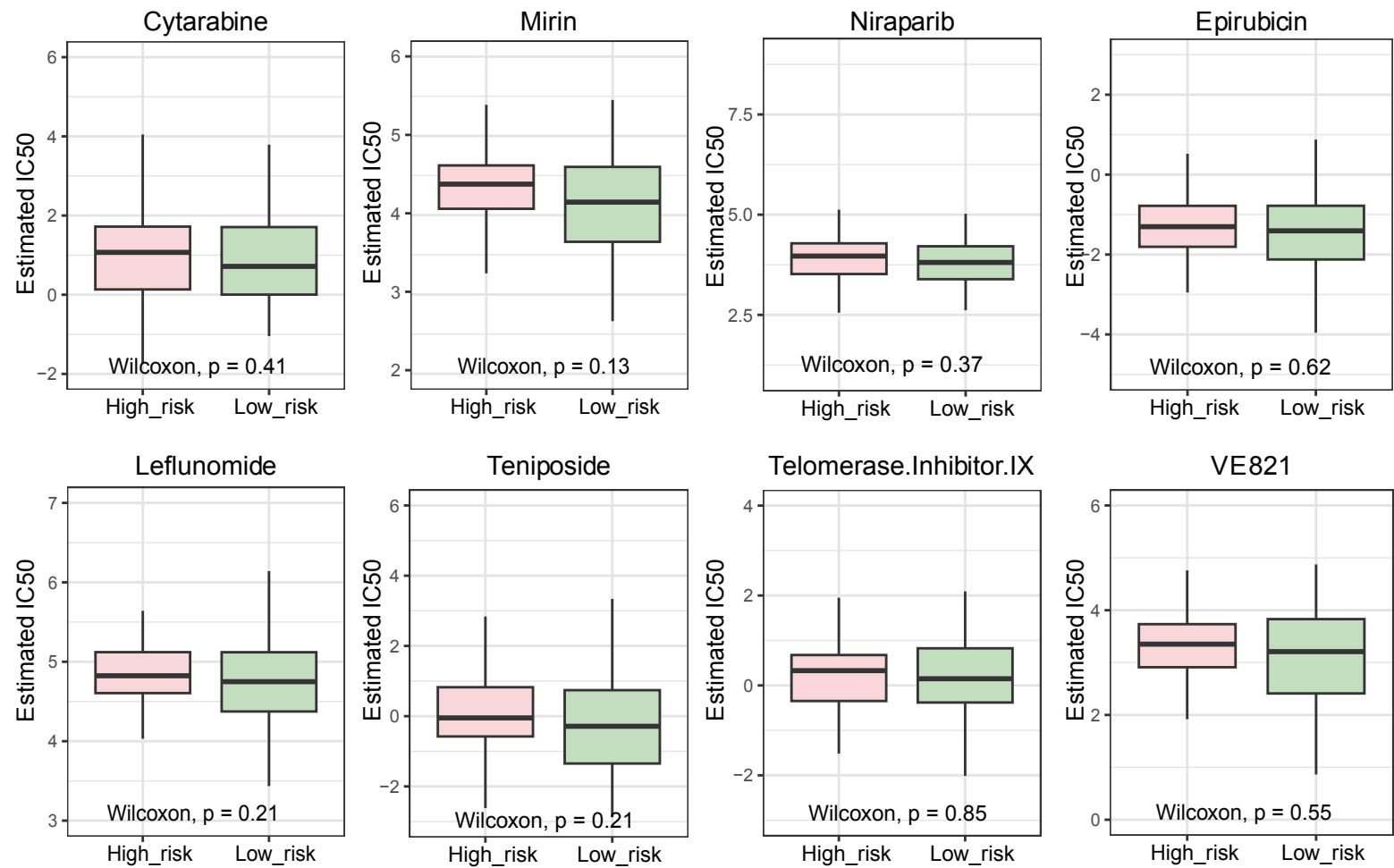

Supplement: Supplementary Figure 1 — Drug sensitivity in high- and low-risk groups. (A) Estimated IC50 of the indicated drugs among two risk groups in the overall patients. (B) Estimated IC50 of the indicated drugs among two risk groups in the TNBC patients. The statistical test was the ‘Wilcoxon test’. [file DataSheet1.pdf]
